# Body Composition Analysis in Obstructive Sleep Apnea: A Cross‐Sectional Study Using Bioelectrical Impedance Analysis

**DOI:** 10.1111/crj.70123

**Published:** 2025-09-18

**Authors:** Mucahit Yetim, Macit Kalçık, Lütfü Bekar, Yusuf Karavelioğlu, Yasemin Arı Yılmaz

**Affiliations:** ^1^ Department of Cardiology Hitit University Faculty of Medicine Çorum Turkey; ^2^ Department of Pulmonary Medicine Hitit University Faculty of Medicine Çorum Turkey

**Keywords:** bioelectrical impedance, body composition, obstructive sleep apnea, total body water, trunk fat

## Abstract

**Introduction:**

Obstructive sleep apnea (OSA) is a prevalent disorder characterized by recurrent upper airway collapse, resulting in intermittent hypoxia and sleep fragmentation. While obesity is a major risk factor, traditional markers such as body mass index (BMI) inadequately reflect the complex interplay of body composition in OSA pathogenesis. This study aimed to investigate the predictive value of body composition parameters assessed by bioelectrical impedance analysis (BIA) for OSA.

**Methods:**

In this cross‐sectional single‐center study, 78 patients diagnosed with OSA by polysomnography (PSG) and 78 age‐, gender‐, and BMI‐matched controls without OSA were analyzed. BIA was used to assess fat distribution, muscle mass, and body water composition. Logistic regression analyses were performed to identify independent predictors of OSA.

**Results:**

Compared to controls, the OSA group had significantly higher lean mass, trunk fat percentage, and total body water. Multivariable logistic regression identified body fat mass (OR = 1.06), visceral fat area (OR = 0.83), and total body water (OR = 1.10) as independent predictors of OSA. Notably, total body water had the strongest association with OSA risk, independent of traditional obesity metrics.

**Conclusion:**

BIA‐derived body composition analysis provides nuanced insights beyond BMI, highlighting the roles of central fat distribution and fluid balance in OSA pathophysiology. These findings underscore the clinical utility of incorporating detailed body composition assessment into the routine evaluation of patients at risk for OSA.

## Graphical abstract

This cross‐sectional study of 156 patients reveals distinct body composition patterns in OSA, with total body water and trunk fat showing stronger associations than BMI. These findings suggest new approaches for OSA risk assessment using bioelectrical impedance analysis.

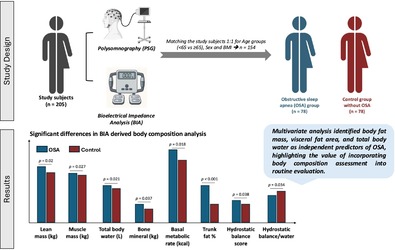

## 1 Introduction

Obstructive sleep apnea (OSA) is a prevalent sleep disorder characterized by recurrent upper airway obstruction during sleep, leading to intermittent hypoxia and sleep fragmentation. The relationship between OSA and obesity is well established, and being overweight is considered a significant risk factor for OSA development and severity [1, 2]. While body mass index (BMI) is commonly used to assess obesity, it fails to capture the nuances of body composition, including fat and muscle mass distribution, which may play a crucial role in OSA pathogenesis [3, 4].

Recent studies revealed that central obesity, particularly visceral fat accumulation and upper body fat distribution, is more strongly associated with OSA than overall obesity [5]. Bioelectrical impedance analysis (BIA) has become a practical, noninvasive method for assessing body composition parameters, including fat mass, muscle mass, and body water distribution [6, 7, 8]. Its affordability, accessibility, and ability to provide a comprehensive analysis make it particularly suitable for clinical and research settings. Several studies have explored the relationships between BIA‐derived body composition parameters and OSA, revealing significant correlations between OSA severity and fat and muscle masses [9, 10]. Although the relationship between fat mass and OSA has been explored, the relationship of muscle mass to OSA, either as a protective or risk‐increasing factor, as well as the relationship between body water distribution and OSA, remains unclear. Additionally, body composition parameters assessed using BIA may be more helpful in diagnosing or determining the severity of OSA. In light of this information, the objective of this study is to assess body composition parameters, such as fat mass, muscle mass, and body water distribution, in patients with OSA and their predictive powers for OSA using BIA.

## 2 Materials and Methods

### 2.1 Population and Sample

**FIGURE 1:**
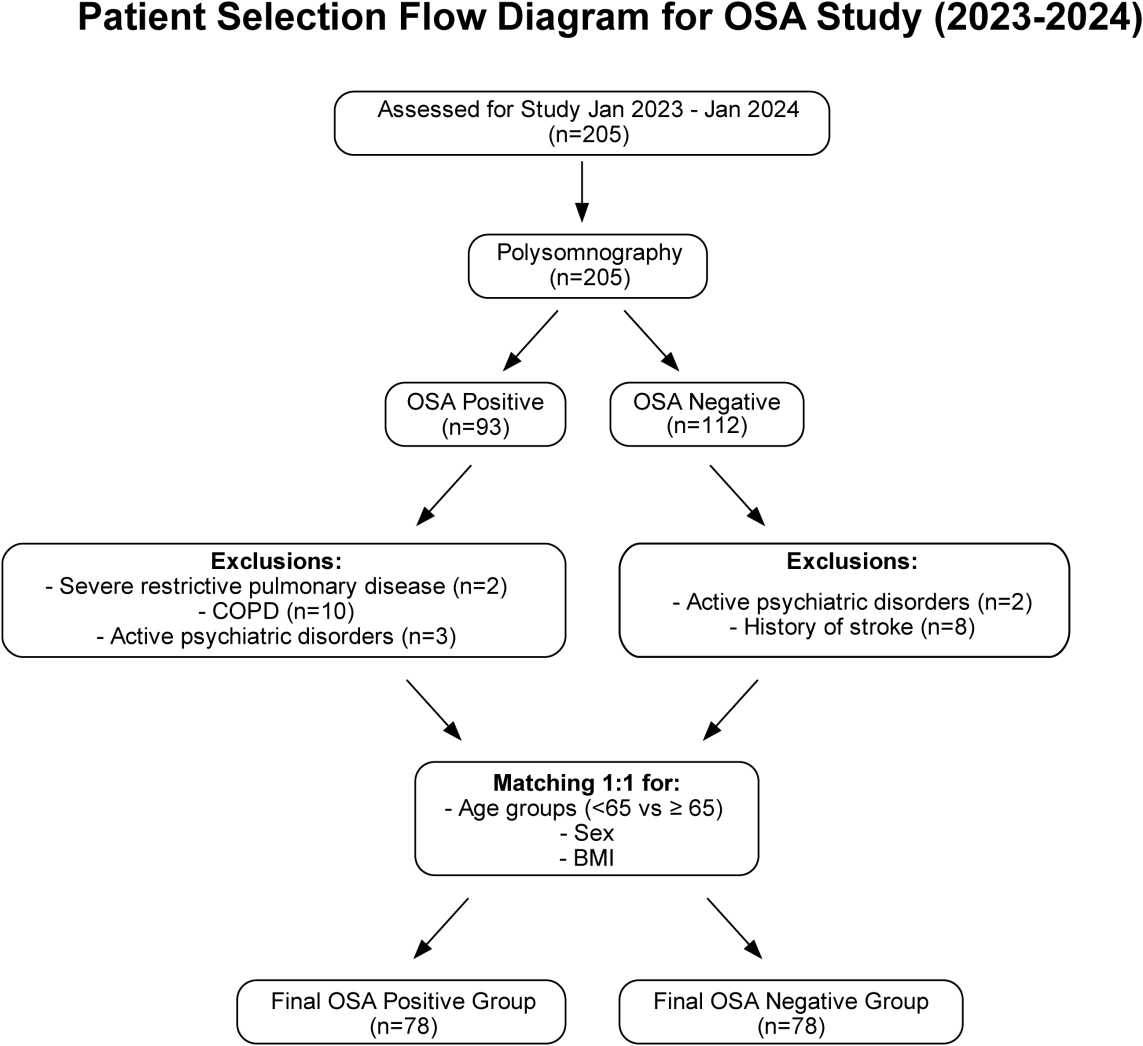
Flow diagram of the inclusion procedure.

This study was designed as a cross‐sectional observational single‐center cohort study. The study population consisted of 205 patients who presented with symptoms suggestive of OSA, including excessive daytime sleepiness, and were referred from cardiology and pulmonary outpatient clinics to the sleep laboratory at the Hitit University Hospital Respiratory Medicine Department between January 2023 and January 2024. The study population was selected from referrals to cardiology and pulmonary clinics to ensure a balanced representation of differing comorbidity profiles. Patients presenting with symptoms suggestive of OSA, including excessive daytime sleepiness, underwent polysomnography (PSG) to confirm the diagnosis of OSA. To maintain the integrity of the study and ensure a homogeneous population for accurate analysis, patients with severe comorbid conditions were excluded from the study. Of the 93 patients whose OSA diagnosis was confirmed by PSG, 10 with chronic obstructive pulmonary disease (COPD), three with active psychiatric disorders, and two with severe restrictive pulmonary disease were excluded from the study. In the end, the OSA group consisted of 78 patients. On the other hand, of the 112 patients who were first admitted to the respiratory sleep clinic and were not diagnosed with OSA, eight with a history of stroke and two with active psychiatric disorders were excluded from the study. Eventually, the control group consisted of 78 patients with an Epworth Sleepiness Scale score of 10 or below, indicating low daytime sleepiness, with age (< 65 years vs. ≥ 65 years), gender, and BMI characteristics matching the OSA group (Figure 1).

### 2.2 Polysomnography Technique

All patients underwent PSG in a controlled environment with stable humidity and temperature, using a Compumedics 44‐channel E‐series PSG device (Australia). The recorded parameters included electroencephalography (EEG), two‐channel electrooculography, electromyography, oronasal airflow, thoracic and abdominal movement, body position, and pulse oximetry parameters. An experienced chest disease specialist evaluated the recorded parameters per the American Sleep Disorders Association criteria [11]. Accordingly, sleep was classified into five stages: Wakefulness (W), non‐rapid eye movement (NREM) stage 1 (N1), NREM stage 2 (N2), NREM stage 3 (N3 or slow‐wave sleep), and rapid eye movement (REM)sleep (R). Scoring was performed in 30‐s epochs, with each stage determined by EEG characteristics and arousals identified by abrupt EEG shifts lasting at least 3 s. Major body movements that obscured EEG signals were documented, with adjustments made as necessary. Respiratory events were classified as apneas, defined by airflow cessation for at least 10 s, or hypopneas, characterized by a 30% airflow reduction lasting 10 s and linked to desaturation or arousal. Periodic breathing patterns, such as Cheyne–Stokes respiration, were also recorded. Sleep apnea severity was assessed using the Apnea‐Hypopnea Index (AHI), defined as the sum of apneas and hypopneas per hour of sleep. An AHI of more than five events per hour of sleep was considered abnormal, and the patient was considered to have a sleep disorder [12]. Patients with an abnormal AHI accompanied by excessive daytime sleepiness were diagnosed with OSA.

The OSA severity was performed in accordance with AHI values as mild (5–14.9 events/h), moderate (15–29.9 events/h), and severe (≥ 30 events/h) (Table 1) [10].

**TABLE 1:** Severity distribution of obstructive sleep apnea based on apnea–hypopnea index in patients with polysomnography‐confirmed OSA.

	OSA group (*n* = 76)
Apnea–hypopnea index (events/h)	33.9 [3.1–97.5]
OSA severity classification	
Mild OSA (AHI 5–14.9 events/h)	9 (11.8)
Moderate OSA (AHI 15–29.9 events/h)	21 (27.6)
Severe OSA (AHI ≥ 30 events/h)	46 (60.5)

*Note:* Data are presented as *n* (%) for categorical variables and median [min–max] for non‐normally distributed variable. Two patients were excluded from analysis (one with missing AHI data, one with AHI < 5 events/h despite OSA diagnosis).

Abbreviations: AHI: apnea–hypopnea index; OSA: obstructive sleep apnea; SD: standard deviation.

### 2.3 BIA and Anthropometric Measurements

Patients underwent BIA on the morning of the day after PSG to assess their fat mass, fat‐free mass, and total body water (TBF‐521, TANITA, Tokyo, Japan). The body analysis scale used was previously validated for patients with OSA [13]. BIA measurements were conducted while the patients were in a standardized standing position and maintaining consistent hydration levels. Patients with metal implants or fluid disturbances were excluded from the BIA analyses. The environment in which the measurements were performed was controlled with stable temperature and precise electrode placement. OSA‐negative patients were scheduled for BIA analyses consistently in a fasting state after rest, avoiding recent fluid intake.

Anthropometric parameters measured included height and weight, and waist circumference. Patients' BMI was calculated by dividing weight (kg) by the square of height (m^2^).

### 2.4 Statistical Analysis

Sample size calculation was performed using G*Power 3.1.9.7 software. Due to the study's cross‐sectional and observational design and the lack of a reference study in the literature that comprehensively examined the relationship between OSA and body composition parameters, the effect size was estimated based on clinical significance. The minimum number of subjects to be included in the study groups was determined as 64 for *α* = 0.05 and *β* = 0.20 (power = 80%), considering a moderate effect size (0.50) for differences in body composition parameters between the study groups. Accordingly, it was decided that at least 76 participants should be included in each study group, assuming that the rate of patients who could drop out of the study and had missing data could be up to approximately 20%.

Post hoc power analysis, which was based on the observed difference in trunk fat percentage between the study groups, revealed that the power of the sample size was 84%.

The results of the statistical analyses were expressed using descriptive statistics, i.e., mean ± standard deviation values in the case of continuous variables determined to conform to the normal distribution, median with minimum and maximum values in the case of continuous variables determined to not conform to the normal distribution, and numbers and percentage values in the case of categorical variables. The normal distribution characteristics of numerical variables were analyzed using Kolmogorov–Smirnov and Anderson–Darling tests for comparisons featuring large sample sizes (*n* ≥ 50) and visual tools such as histograms and Q‐Q (quantile‐quantile) plots. Pearson's chi‐square test was used to compare categorical variables between the groups in 2 × 2 tables with expected cells of five or more, as it provides more reliable results with large sample sizes. Additionally, in comparing the differences in numerical variables between two independent groups, the independent samples *t*‐test was used for numerical variables determined to conform to the normal distribution, and the Mann–Whitney *U* test was used for numerical variables determined to not conform to the normal distribution. The multivariate logistic regression model was developed using a systematic variable selection approach. Variables found to significantly (*p* < 0.20) predict OSA in the univariate model were further analyzed using the multivariate model. Before being included in the multivariate analysis, the multicollinearity of the variables was assessed using the variance inflation factor (VIF). Accordingly, variables with a VIF of > 5 were excluded from the analysis. Subsequently, a backward stepwise selection procedure was applied with inclusion and exclusion criteria set at *p* < 0.10 and *p* < 0.05, respectively. Model building was based on the purposeful selection strategy described by Hosmer and Lemeshow, which envisages the inclusion of clinically relevant variables regardless of their statistical significance. Potential interaction terms between variables, particularly between body water parameters and fat distribution metrics, were tested, and those with a significance level of *p* ≤ 0.05 were included in the analysis.

Missing data analysis revealed that < 5% of data points were missing across all variables, satisfying the assumption of missing completely at random (MCAR), as confirmed by Little's test (*p* > 0.05). Complete case analysis was deemed appropriate given the minimal missing data and MCAR pattern. However, sensitivity analyses using multiple imputations (with 20 imputed datasets) were performed to confirm the robustness of our findings. The final model's stability was assessed through bootstrapping with 1000 replications. The initial model included only the constant term, and through the variable selection process, the model's explanatory power was enhanced, resulting in an accuracy rate of approximately 66%.

Model fit measurements revealed a deviance value of 197, with an Akaike Information Criterion (AIC) of 205 and a Bayesian Information Criterion (BIC) of 217. *R* ^*2*^ measures calculated for model explanatory power are as follows: McFadden *R* ^*2*^ = 0.0911, Cox and Snell *R* ^*2*^ = 0.119, Nagelkerke *R* ^*2*^ = 0.158, and Tjur *R* ^*2*^ = 0.119. The overall model test revealed the significance of the model (*χ* ^2^ = 19.7, df = 3, *p* < 0.001), as confirmed by the Hosmer–Lemeshow goodness‐of‐fit test (*p* > 0.05).

The cut‐point analysis revealed 0.5 as the optimal threshold balancing sensitivity and specificity. According to the classification table, the accurate classification rates were 65.4% and 66.7% for OSA and control groups, respectively, with an overall accuracy of 66%. Performance metrics included accuracy (66%), specificity (66.7%), sensitivity (65.4%), and area under the receiver operating characteristic (ROC) curve (AUC, 0.700) for moderate discriminative power.

The ROC curve analysis indicated that the established model had moderate discriminative power. Only one case (Case 77) was misclassified, as evidenced by a standardized residual more significant than 2, indicating a good model fit overall.

Statistical analyses were conducted using Jamovi project 2.3.28 (Jamovi, version 2.3.28.0, 2023, retrieved from https://www.jamovi.org) and JASP 0.19.0 (Jeffreys' Amazing Statistics Program, version 0.19.0, 2024, retrieved from https://jasp‐stats.org) software packages. Probability (*p*) statistics of ≤ 0.05 were deemed to indicate statistical significance.

## 3 Results

The mean age of the OSA group was significantly lower than the control group (52.0 ± 10.5 vs. 57.4 ± 9.7 years, *p* = 0.001). However, there was no significant difference between the groups in terms of the number of patients included in the < 65 and ≥ 65 age groups. There was also no significant difference between the groups in other demographic and anthropometric characteristics, including gender and BMI (Table 2). On the other hand, the groups differed significantly in terms of comorbidities. Accordingly, the rates of patients with hypertension, dyslipidemia, and coronary artery disease were significantly lower in the OSA group than in the control group (38.5% vs. 59.0%, *p* = 0.016; 14.1% vs. 64.1%, *p* < 0.001; and 5.1% vs. 29.5%, *p* < 0.001; respectively).

**TABLE 2:** Demographic characteristics, anthropometric measurements, and comorbidities of the study population comparing Control and OSA groups.

	Control group (*n* = 78)	OSA group (*n* = 78)	*p*
Age, years^†^	57.4 ± 9.7	52.0 ± 10.5	**0.001** ***
Age, years^‡^			
	59 (75.6)	67 (85.9)	0.155*
≥ 65	19 (24.4)	11 (14.1)	
Age, years^‡^			
< 40	3 (3.8)	5 (6.4)	0.719*
≥ 40	75 (96.2)	73 (93.6)	
Gender^‡^			
Male	48 (61.5)	46 (59.0)	0.870*
Female	30 (38.5)	32 (41.0)	
Height, cm^§^	163.0 [147.0–183.0]	165.0 [146.0–188.0]	0.787**
Weight, kg^§^	85.7 [57.1–125.9]	89.0 [53.0–137.4]	**0.017** **
Body mass index (BMI)^§^	31.6 [20.2–49.2]	33.3 [19.5–56.6]	0.081**
Waist circumference (cm)	106 [83–126]	108 [80–128]	0.777**
Smoking status, yes^‡^	25 (32.1)	24 (30.8)	0.999*
Hypertension, present^‡^	46 (59.0)	30 (38.5)	**0.016** *
Diabetes, present^‡^	24 (30.8)	22 (28.2)	0.861*
Dyslipidemia (hyperlipidemia), present^‡^	50 (64.1)	11 (14.1)	**< 0.001** *
Coronary artery disease (CAD), present^‡^	23 (29.5)	4 (5.1)	**< 0.001** *
Chronic kidney failure, present^‡^	2 (2.6)	0 (0.0)	0.497*

*Note:* Significant *p*‐values (*p* < 0.05) are shown in bold.

Abbreviations: cm: centimeter, kg: kilogram, OSA: obstructive sleep apnea.

* Pearson Chi‐Square test.

** Mann–Whitney *U* test.

*** Independent samples *t*‐test.

*n* (%).

Mean ± standard deviation.

Median [min–max].

In terms of body composition parameters, the OSA group had significantly higher mean lean mass (60.5 ± 10.1 kg vs. 56.8 ± 9.4 kg, *p* = 0.020), mean muscle mass (57.5 ± 9.7 kg vs. 54.3 ± 8.5 kg, *p* = 0.027), mean total body water (43.3 ± 7.5 L vs. 40.8 ± 6.2 L, *p* = 0.021), bone mineral content (3.1 kg vs. 2.9 kg, *p* = 0.037), basal metabolic rate (1806.5 kcal vs. 1713.5 kcal, *p* = 0.018), and mean trunk fat percentage in particular (31.0% vs. 14.4%, *p* < 0.001) than the control group (Table 3). There was no significant difference between the groups in other body composition parameters, including body fat percentage, fat mass, metabolic age, protein content, visceral fat ratio, total body water percentage, hydrostatic insulin score, and trunk muscle mass (*p* > 0.05).

**TABLE 3:** Comparison of body composition parameters and metabolic measurements between Control and OSA Groups.

Parameters	Control group (*n* = 78)	OSA group (*n* = 78)	*p*
Body fat percentage, %^†^	32.2 ± 7.7	32.7 ± 9.4	0.726*
Fat mass, kg^§^	27.2 [5.3–60.6]	29.1 [11.0–62.4]	0.233**
Lean mass, kg^†^	56.8 ± 9.4	60.5 ± 10.1	**0.020** *
Muscle mass, kg^†^	54.3 ± 8.5	57.5 ± 9.7	**0.027** *
Metabolic age, years^§^	64.0 [25.0–85.0]	59.0 [23.0–80.0]	0.053**
Bone mineral content, kg^§^	2.9 [2.1–3.7]	3.1 [1.9–4.1]	**0.037** **
Protein, kg^†^	13.8 ± 2.2	14.2 ± 2.3	0.281*
Basal metabolic rate (kJ)^§^	7169.5 [5330.0–9330.0]	7558.0 [66.9–10648.0]	**0.024** **
Basal metabolic rate (kcal)^§^	1713.5 [141.0–2230.0]	1806.5 [1174.0–2545.0]	**0.018** **
Visceral fat ratio^§^	13.0 [3.0–32.5]	13.0 [4.0–24.0]	0.432**
Total body water, L^†^	40.8 ± 6.2	43.3 ± 7.5	**0.021** *
Total body water percentage, %^§^	48.8 [15.2–66.2]	49.2 [35.2–62.4]	0.821**
Trunk muscle mass, kg^†^	30.5 ± 4.2	31.7 ± 4.5	0.091*
Trunk fat percentage, %^†^	14.4 ± 3.7	31.0 ± 6.2	**< 0.001** *

*Note:* Significant *p*‐values (*p* < 0.05) are shown in bold.

Abbreviations: kg: kilogram, L: liter, OSA: obstructive sleep apnea.

* Independent samples *t*‐test.

** Mann–Whitney *U* test.

Mean ± standard deviation.

Median [min–max].

**FIGURE 2:**
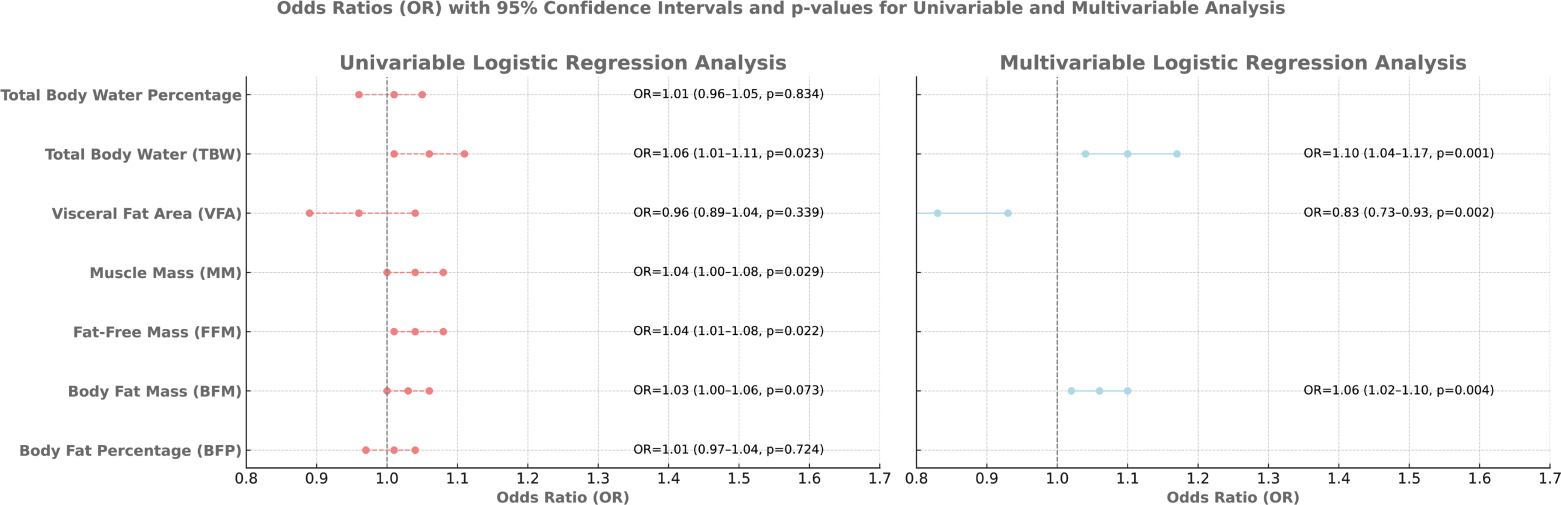
Forest plot comparing univariable (left panel, red) and multivariable (right panel, blue) logistic regression analyses of body composition parameters. Values show odds ratios (OR) with 95% confidence intervals and *p*‐values. Vertical dashed line at OR = 1.0 represents the null hypothesis.

The univariate analysis revealed that fat‐free mass (OR = 1.04, *p* = 0.022), muscle mass (OR = 1.04, *p* = 0.029), and total body water (OR = 1.06, *p* = 0.023) were significantly associated with OSA, while body fat percentage, body fat mass, visceral fat area, and total body water percentage were not (*p* > 0.05) (Table 4). The multivariate analysis revealed three independent predictors of OSA: body fat mass (odds ratio [OR] = 1.06, 95% confidence interval [CI]: 1.02–1.10, *p* = 0.004), visceral fat area (OR = 0.83, 95% CI: 0.73–0.93, *p* = 0.002), and total body water (OR = 1.10, 95% CI: 1.04–1.17, *p* = 0.001). Total body water percentage was found to have the strongest positive association with OSA after the adjustment was made for confounders (Figure 2).

**TABLE 4:** Univariable and multivariable logistic regression analyses for predicting obstructive sleep apnea based on body composition parameters.

Logistic regression analysis predicting the likelihood of obstructive sleep apnea	OR (univariable)	OR (multivariable)
Body fat percentage, %	1.01 (0.97–1.04, *p* = 0.724)	—
Body fat mass, kg	1.03 (1.00–1.06, *p* = 0.073)	1.06 (1.02–1.10, ***p* = 0.004**)
Fat‐free mass, kg	1.04 (1.01–1.08, ***p* = 0.022**)	—
Muscle mass, kg	1.04 (1.00–1.08, ***p* = 0.029**)	—
Visceral fat area, m^2^	0.96 (0.89–1.04, *p* = 0.339)	0.83 (0.73–0.93**, *p* = 0.002**)
Total body water, L	1.06 (1.01–1.11, ***p* = 0.023**)	1.10 (1.04–1.17**, *p* = 0.001**)
Total body water percentage, %	1.01 (0.96–1.05, *p* = 0.834)	—

*Note:* Values are presented as odds ratio (OR) with 95% confidence intervals and *p*‐values. Significant associations (*p* < 0.05) in multivariable analysis are included in the final model. Significant *p*‐values (*p* < 0.05) are shown in bold. Model performance metrics: Overall accuracy: 66%, sensitivity: 65.4%, specificity: 66.7%, AUC: 0.700. Model fit was confirmed by Hosmer–Lemeshow test (*p* > 0.05). The model exhibited moderate discriminative power (*χ* ^2^ = 19.7, df = 3, *p* < 0.001, Nagelkerke *R* ^*2*^ = 0.158).

## 4 Discussion

The relationship between obesity and OSA presents a classic “chicken and egg” dilemma, with each condition perpetuating the other. Our study revealed that the OSA group had significantly higher levels of muscle mass and total body water, as well as trunk fat percentage compared to the control group. These findings align with Matsumoto et al.'s findings, who reported that the accumulation of more fat in muscle tissues is a significant risk factor for OSA [9]. As a matter of fact, while obesity status could not explain the occurrence of OSA in one but not the other individual with the same BMI, altered fat distribution patterns could [3, 5]. While obesity increases the risk of airway collapse by narrowing the upper airway due to excessive fat accumulation around the neck, OSA leads to weight gain by disrupting the hormonal balance, resulting in decreased physical activity and higher caloric intake. The fact that visceral fat contributes to airway collapse and supports inflammatory mediators may explain the relationship between fat distribution and OSA severity. Thus, it is central obesity, rather than general obesity, that plays a key role in the pathogenesis of OSA [6, 10]. The above‐mentioned findings are consistent with previous studies showing that fat accumulation around the upper airway worsens OSA progression and severity [9, 14, 15].

Interestingly, despite our finding that trunk fat percentage was significantly more elevated in the OSA group than in the control group, multivariate analysis revealed a more complex relationship between visceral fat area and OSA. The negative relationship of visceral fat area with OSA suggests that the role of fat distribution in OSA may not be as straightforward as previously thought. In line with Zhang et al.'s findings [16], we found that muscle mass and fat‐free mass were significantly increased in patients with OSA, suggesting that other physiological factors such as upper airway anatomy, neuromuscular control, or genetic predispositions may be a factor in the effect of visceral fat on OSA [17, 18].

Adipose tissue inflammation and adipokine profiles significantly affect obesity‐related metabolic disorders. Obesity causes chronic low‐grade inflammation in adipose tissue due to dysfunctional adipocytes that secrete pro‐inflammatory adipokines, attracting immune cells such as macrophages and worsening inflammation [19]. This inflammatory state disrupts insulin signaling, with cytokines such as tumor necrosis factor‐alpha (TNF‐α) and interleukin 6 (IL‐6) leading to insulin resistance and complications such as type 2 diabetes [20]. Considering that the imbalance in adipokines, manifested especially by high leptin and low adiponectin levels, exacerbates metabolic dysfunction, it has been suggested that the leptin–adiponectin ratio may be a potential metabolic risk biomarker [6, 20]. While pro‐inflammatory M1 macrophages increase inflammation, M2 macrophages exert a protective effect. The mechanical stress from adipose tissue expansion and hypoxia activates inflammatory pathways through hypoxia‐inducible factors that promote inflammatory gene expression and macrophage recruitment [21]. These interconnected processes underscore adipose tissue inflammation and adipokine imbalance's complex role in obesity‐related metabolic disorders.

An interesting finding of our study is the strong relationship between total body water percentage and OSA severity. Increased total body water in patients with OSA may reflect fluid changes that exacerbate airway narrowing, especially when in the supine position. This fluid redistribution hypothesis is supported by Lovin et al.'s findings that body water in patients with OSA is associated with disease severity [10]. Body water distribution is altered in OSA. Nocturnal pulmonary hypertension, increased atrial natriuretic peptide release, altered renin–angiotensin–aldosterone activity, and high levels of endothelin may explain the altered fluid distribution in OSA featuring nocturnal polyuria, peripheral edema, and hemoconcentration [22]. Our finding that common metabolic indicators such as body fat percentage and fat mass have a weaker relationship with OSA compared to central and fluid‐related metrics indicates that detailed body composition analysis may be more useful in clinical assessments than analyses based on general obesity parameters such as BMI.

Another interesting finding of our study is the relationship we observed between lean mass and OSA. The increase in lean mass in patients with OSA, which is generally thought to be beneficial to metabolic health, may indicate a compensatory mechanism or reflect muscle hypertrophy in response to chronic hypoxia and increased respiratory effort [16, 18]. This finding underscores the need for a nuanced understanding of changes in body composition in OSA and how they may reflect both pathophysiological adaptations and risk factors.

The highlight of our study is that it demonstrated the relationship between OSA and body composition parameters obtained using BIA. In particular, the strong relationships found between OSA and body fat distribution and body water parameters suggest that these parameters can be used in OSA risk assessment. In sum, our study emphasizes the dynamic nature of body composition in OSA and reveals that not a single parameter, but many factors should be evaluated together.

A notable strength of our study is the exclusion of patients with COPD, which eliminates the potential confounding effects of COPD–OSA overlap syndrome. This condition is characterized by distinct pathophysiological mechanisms, more severe nocturnal oxygen desaturation, different body shapes and compositions, and unique comorbidity profiles compared to OSA alone [23]. In addition to contributing to the global literature, our findings provide valuable region‐specific data on the relationship between OSA and body composition. Given that demographic characteristics, lifestyle factors, and comorbidity patterns vary across populations, such local evidence is essential for developing tailored prevention, screening, and management strategies.

## 5 Limitations of the Study

This study has several limitations. First, its cross‐sectional design precludes establishing a causal relationship between changes in body composition and OSA. Second, the single‐center design may have introduced selection bias. Patients referred from cardiology clinics predominantly exhibited cardiovascular comorbidities, while those referred from pulmonary clinics had a higher prevalence of respiratory conditions, reflecting the typical patient profiles of each specialty. Consequently, the recruitment strategy may have influenced the distribution of comorbidities in our sample. Third, the inclusion of participants with a wide range of ages and BMI values may limit the generalizability of our findings. Fourth, the absence of data on OSA duration and prior treatments could have affected the interpretation of body composition differences. Fifth, although body composition was assessed using a validated BIA method, techniques such as dual‐energy X‐ray absorptiometry (DEXA) and computed tomography (CT) provide more precise and detailed measurements. While BIA measurements were standardized by performing them in the morning after PSG, evaluating body composition at a single time point does not capture diurnal variations. Finally, although our predictive model demonstrated moderate discriminative ability (AUC = 0.700), its accuracy, sensitivity, and specificity were limited, which may restrict its applicability as a standalone clinical screening tool for OSA.

In conclusion, body fat mass, visceral fat area, and total body water were found to be independent risk factors for OSA, with total body water having the strongest relationship with OSA. The significant difference in body fat percentage between patients with and without OSA emphasizes the importance of fat distribution patterns rather than general obesity. The differences found in BIA results between patients with and without OSA suggest that body composition analysis could be an essential tool in evaluating patients with OSA.

## Author Contributions

All authors have made significant contributions to this study. Mucahit Yetim: Study design, data collection, analysis, interpretation, and writing. Macit Kalçık: Study design, clinical evaluation, data analysis, and critical revision. Lütfü Bekar: Data collection, data analysis, and contribution to the writing process. Yusuf Karavelioğlu: Study concept, clinical evaluation, and critical revision. Yasemin Arı Yılmaz: Data collection, polysomnographic evaluation, and methodological consultation. All authors have reviewed and approved the final version of the manuscript.

## Ethics Statement

This study was approved by the Ethics Committee of Hitit University Faculty of Medicine (Date: 09.04.2025, Decision No: 2025‐56). Written informed consent was obtained from the parents or legal guardians of all participants. The study was conducted in accordance with the ethical considerations outlined in the Declaration of Helsinki and the principles of Good Clinical Practice with due respect to the rights and dignity of all participants.

## Conflicts of Interest

The authors declare no conflicts of interest.

## Data Availability

The data that support the findings of this study are available from the corresponding author upon reasonable request.
